# The basis for cosmic ray feedback: Written on the wind

**DOI:** 10.1063/1.4984017

**Published:** 2017-05-24

**Authors:** Ellen G. Zweibel

**Affiliations:** Department of Astronomy, University of Wisconsin-Madison, Wisconsin 53706, USA and Department of Physics, University of Wisconsin-Madison, Wisconsin 53706, USA

## Abstract

Star formation and supermassive black hole growth in galaxies appear to be self-limiting. The mechanisms for self-regulation are known as *feedback*. Cosmic rays, the relativistic particle component of interstellar and intergalactic plasma, are among the agents of feedback. Because cosmic rays are virtually collisionless in the plasma environments of interest, their interaction with the ambient medium is primarily mediated by large scale magnetic fields and kinetic scale plasma waves. Because kinetic scales are much smaller than global scales, this interaction is most conveniently described by fluid models. In this paper, I discuss the kinetic theory and the classical theory of cosmic ray hydrodynamics (CCRH) which follows from assuming cosmic rays interact only with self-excited waves. I generalize CCRH to generalized cosmic ray hydrodynamics, which accommodates interactions with extrinsic turbulence, present examples of cosmic ray feedback, and assess where progress is needed.

## I. INTRODUCTION

Cosmic rays are pervasive in galaxies. They have been studied *in situ* at Earth for over a century, and remotely, primarily through their radio and *γ*-ray emissions, since the 1950s and 1960s, respectively, making them important probes of the interstellar medium.

In Milky Way-like galaxies, the energy density in cosmic rays is approximately in equipartition with the magnetic and turbulent energy densities. Therefore, the role of cosmic rays in the dynamics and energy balance of interstellar gas, and, by implication, the aspects of galaxy evolution that are driven by the gas, such as star formation, change of chemical composition, and growth and maintenance of galactic magnetic fields, is of considerable interest. In particular, cosmic rays are implicated in *feedback*, the mechanisms by which star formation and supermassive black hole growth regulate themselves by deposition of energy and momentum in the ambient medium.

Cosmic rays interact with the thermal gas both collisionally and collisionlessly. Relatively low energy (2–10 MeV) cosmic rays are an important source of collisional ionization and heating in the interstellar medium. The protons of a few GeV, where most of the cosmic ray energy density resides, are essentially collisionless. Their coupling is mediated by the ambient magnetic field and by kinetic scale waves and instabilities. The plasma physics of this coupling, and a few examples of why it matters, are the subject of this article.

The first studies of cosmic ray coupling concerned transport, i.e., diffusion of cosmic rays through fluctuating magnetic fields.^1–6^ Many aspects of this topic remain unresolved, particularly the extent to which cosmic rays can be transported across magnetic fieldlines.^7,8^ In the late 1960s, it was realized that the waves that scatter cosmic rays can be generated by the cosmic rays themselves through a kinetic instability,^9,10^ and that this could lead to astrophysically important energy and momentum exchange between the cosmic rays and the background.^11^ An elegant set of fluid equations describing energy and momentum exchange according to this self-confinement picture was developed in the 1980s, originally to model cosmic ray acceleration by collisionless shocks in a self-consistent way,^12–14^ and later to model cosmic ray driven galactic winds.^15^ We call this set of equations “classical cosmic ray hydrodynamics” (CCRH). Since cosmic rays affect the background medium, including the turbulence, through which they propagate, this form of cosmic ray transport is sometimes said to be nonlinear.

In this paper, we return to the early theories of cosmic transport, which make no assumptions as to how the waves are generated, and show that they lead to a more general set of fluid equations, which we call “generalized cosmic ray hydrodynamics” (GCRH). The paper is organized as follows. In Sec. II, we briefly review salient properties of galactic magnetic fields and cosmic rays. In Sec. III, we delve briefly into the wave–particle interactions which underlie the fluid theory for cosmic rays, and derive the Fokker-Planck equation on which the fluid theory is based. The extended fluid treatment and some approximations to it, which constitute GCRH, are developed in Sec. IV, which is the technical heart of the paper. In Sec. V, we discuss some applications which show that the treatment of cosmic rays makes a difference.

Cosmic ray hydrodynamics and the kinetic theory on which it is based have also been applied to galaxy clusters, for which there are some puzzling observations. In Sec. VI, we develop some further generalizations of cosmic ray hydrodynamics to accommodate conditions in the cluster environment. Section VII is a summary and discussion.

The focus of this paper is fairly tight, and the reader with more general interests may wish to consult other work on cosmic rays. For excellent recent overviews of cosmic rays, see Refs. 16 and 17; for a review that focusses on the ultrahigh energy component, see Ref. 18. Short reviews of acceleration and propagation with a more theoretical emphasis are given in Refs. 19–21, while Ref. 22 is a pedagogical reference. Yet, such is the level of activity in this field that in order to keep the paper of manageable length it has been necessary to omit many interesting subtopics, and references.

## II. SELECTED PROPERTIES OF THE GALACTIC MAGNETIC FIELD AND COSMIC RAYS

The properties of the magnetic fields in spiral galaxies, including the Milky Way, were recently reviewed in Ref. 23. The Milky Way magnetic field is coherently directed on scales of at least several kpc. It is parallel to the Galactic plane and nearly azimuthal, and occupies a 2–4 kpc thick layer. In addition to the coherent component, there is a randomly oriented component that is at least as strong, such that the overall field strength in the solar neighborhood is about 5 *μ*G.

The Galactic cosmic ray spectrum extends from about 10^6^ eV up to at least 10^20^ eV and is usually represented as a broken power law for energies above about 10^9^ eV, with a rollover at lower energies. The total energy density in cosmic rays is about 1 eV cm^−3^, and the mean particle energy is about 3 × 10^9^ eV. The corresponding number density is thus about 3 × 10^−10 ^cm^−3^, much lower than the mean interstellar gas density of about 1 cm^−3^. One consequence of this low density is that although about 98%–99% of cosmic rays are positive ions, their net current can be compensated by a highly subthermal drift of interstellar electrons, which we will assume to be the case. Cosmic rays at the mean energy are mostly protons, and the slope of the spectrum in the GeV to PeV energy range is *ϵ*^−2.6^ or *p*^−4.6^, where *ϵ* and *p* are relativistic energy and momentum, respectively.

Cosmic rays are highly enriched in the elements LiBeB, which are thought to result from collisions between cosmic ray CNO nuclei and interstellar hydrogen. From this hypothesis, it is inferred that the cosmic rays detected at Earth have passed through about 5 gm cm^−2^ since they were accelerated, and that the grammage decreases with energy as *ϵ*^−0.3^–*ϵ*^−0.6^. From these results, and from the abundance ratio of the radioactive and stable isotopes ^10^Be and ^9^Be, it appears that GeV cosmic rays are confined to the galaxy for about 2 × 10^7^ year, that the confinement time decreases as *ϵ*^0.3^–*ϵ*^0.6^ and the mean density of the confinement volume is about 0.1 cm^−3^, lower than that at the Galactic midplane but representative of the ionized gas layer that extends 1–2 kpc above the Galactic plane, similar to the half thickness of the magnetic field layer.^24^

Within the solar system, the gyroradii of cosmic rays below about 1 TeV are small enough that their orbits are significantly perturbed by the heliospheric magnetic field, making their spatial anisotropy in the interstellar medium difficult to measure. At energies at which the anisotropy can be reliably measured, it is low—less than 10^−3^ at 1 TeV and larger, but still less than 10^−2^, at 1 PeV.

From the long confinement times and weak anisotropy of cosmic rays, the respective decrease and increase of these properties with energy, and the known orientation and strength of the Galactic magnetic field, we infer that cosmic rays by and large propagate diffusively along magnetic field lines, with a scattering mean free path of about 1 pc at GeV energies, increasing as *ϵ*^0.3^–*ϵ*^0.6^ and a scattering process that approximately preserves particle energy.

## III. KINETIC THEORY

Gyroresonant scattering by small amplitude hydromagnetic waves fulfills all the required conditions for a scattering mechanism and is generally considered the dominant scattering process. The resonance condition for a particle with pitch angle cosine μ≡v·B/vB, interacting with a wave of frequency *ω* and parallel wavenumber $k_{∥}$, is

$$\omega -k_{∥}v\mu \pm \omega_{c}=0,$$ (1)

where *ω_c_* = *ZeB*/*γmc *=* ZeBv*/*cp* is the relativistic gyrofrequency, $\omega \equiv \omega_{A}=k_{∥}v_{A}$ for an Alfven wave, and the ± signs denote right and left circular polarization, respectively. In the interstellar medium, *v_A_* is typically tens of km/s, so we will frequently treat *v_A_*/*v* ∼ *v_A_*/*c* as a small parameter. Using the definition of *ω_c_* and dropping ω≪kv, we see that there is a minimum momentum *p*_1_(*k*) that can resonate with a wave of wavenumber *k*:

$$p_{1}(k)\equiv \frac{ZeB}{ck}.$$ (2)

We will assume proton cosmic rays (*Z* = 1) in all future expressions and estimates.

The rate of diffusion in pitch angle θ=arccosμ due to successive uncorrelated scatterings by resonant waves of wavenumber *k* and amplitude *B*_1__*k*_ on a background field *B* can be estimated by assuming that each encounter results in a deflection of order Δ*θ* ∼ *B*_1__*k*_/*B* and that the rate of encounters is *ω_c_*. The resulting rate of diffusion in *μ*, or scattering frequency *ν*, is then

$$\frac{\left\langle {\left(\Delta \mu \right)}^{2}\right\rangle }{\Delta t}∼\frac{\pi }{2}\omega_{c}\left(1-\mu^{2}\right){\left(\frac{B_{1k}}{B}\right)}^{2}$$ (3)

(the factor of *π*/2 results from a more exact calculation^6^). To order of magnitude, the resulting scattering frequency yields the appropriate confinement time for GeV cosmic rays if *B*_1__*k*_/*B* is of order 10^−3^.

From Faraday's Law, the wave electric and magnetic field amplitudes are related by $E_{1k}/B_{1k}∼\omega /ck∼v_{A}/c\ll 1$; therefore, the rate of diffusion in *p* is smaller than the rate of diffusion in *μ* by a factor of order (*v_A_*/*c*)^2^. As small as the energy exchange rate is, it contributes to wave growth or damping, depending on whether the ensemble of cosmic rays is drifting faster or slower than the wave speed.

The gyroresonant instability of hydromagnetic waves, also called the streaming instability,^10^ is derived from standard linearized Vlasov perturbation theory, leading to the expression for the growth or damping rate Γ_*c*_ of parallel propagating hydromagnetic waves

$$\Gamma_{c}(\omega ,k)=\frac{\pi^{2}q^{2}}{2}\frac{v_{A}^{2}}{c^{2}}\int \delta (\omega -kv\mu \pm \omega_{c})v(1-\mu^{2})A[f,k,\omega ]p^{2}dpd\mu ,$$ (4)

where *f* is the gyro-averaged zero order cosmic ray distribution function and

$$A[f,k,\omega ]\equiv \frac{\partial f}{\partial p}+\left(\frac{kv}{\omega }-\mu \right)\frac{1}{p}\frac{\partial f}{\partial \mu }.$$ (5)

Equations (4) and (5) are written in the rest frame of the background fluid. Oblique waves are also unstable, but the growth rate is maximal for parallel propagation; a general expression for Γ_*c*_ valid for Alfven and fast mode waves at arbitrary propagation angles is given in Ref. 10. In deriving Eq. (4), it is assumed that the cosmic ray density is so low that their only significant contribution to the plasma dispersion relation is growth or damping. The full cosmic ray contribution to the plasma dielectric is important for large cosmic ray fluxes^25,26^ and can lead to rapid growth of the magnetic field near strong shocks.^27,28^

The sign of A determines whether the wave is unstable (A>0) or damped (A<0). Since *∂f*/*∂p* < 0 for the steady state *f*, instability can only occur for sufficiently large ∂f/∂μ. However, due to the large multiplier factor *kv*/*ω* ∼ *c*/*v_A_*, even a small anisotropy of order *v_A_*/*c* destabilizes the wave. It can be shown that A is *∂f*/*∂μ* in a frame moving with speed *ω*/*k*, so instability corresponds to positive (negative) anisotropy in a frame moving with a forward (backward) going wave. In other words, waves propagating in the same direction as the streaming cosmic rays are unstable if the cosmic rays stream faster than the waves. While linearly polarized waves are only destabilized by streaming anisotropy, circularly polarized waves can be destabilized by pressure anisotropy of order *v_A_*/*c*. However, we will not discuss this further here.

The growth rate Γ_*cr*_ from Eq. (4) can be written as

$$\Gamma_{c}∼\frac{\pi }{4}\omega_{cp}C\frac{n_{c}(>p_{1})}{n_{i}}\left(\frac{v_{D}}{v_{A}}-1\right),$$ (6)

where *ω_cp_* is the nonrelativistic proton gyrofrequency, *C* is a constant of order unity equal to (*α* – 3)/(*α* – 2) for a *p*^–*α*^ spectrum, *p*_1_ is defined in Eq. (2), *n_c_*(> *p*_1_) is the number density of cosmic rays of any *μ* that can resonate with a given wave, *n_i_* is the thermal ion density, and *v_D_* is the velocity of the frame in which the cosmic rays are isotropic (it is assumed that there exists such a frame). For a *p*^–*α*^ spectrum, $n_{c}(>p_{1})\propto p_{1}^{3-\alpha }$, so waves that scatter high energy cosmic rays have lower growth rates than waves that scatter low energy cosmic rays.

The discussion so far is independent of the origin of the waves that scatter the cosmic rays. The gyroresonant interaction that gives rise to the streaming instability—or its counterpart, wave damping—always acts at some level. In the so-called self-confinement model that underlies CCRH, the streaming instability is the dominant source of waves. In the opposite limit, which we call the extrinsic turbulence model, the waves are driven almost entirely by other processes. There is a continuum of models between these two limits, which is encompassed by GCRH. While a good case can be made that self-confinement is the right theory for fluid models, which are dominated by cosmic rays in the GeV range, it was appreciated early in the development of self-confinement theory that due to the decrease in Γ_*c*_ with decreasing *k* (or increasing *p*_1_), self-confinement must break down above some critical energy,^29^ above which the scattering waves have another origin. Subsequent work has expanded upon this idea.^30,31^

The next step in deriving the fluid theory is to calculate the reaction of an ensemble of small amplitude, randomly phased waves on the cosmic rays. This is usually done with quasilinear theory, and includes only scattering by resonant waves. Denoting the perturbation to the distribution function by *f*_1_ gives

$$\frac{\partial f}{\partial t}+v\cdot \nabla f=-\left\langle \frac{q}{m}\left(E_{1}+\frac{v\times B_{1}}{c}\right)\cdot \nabla_{p}f_{1}\right\rangle ,$$ (7)

where the angle brackets denote averaging over wave period and phase. Inserting *f*_1_ obtained from linear perturbation theory yields a diffusion equation in momentum space

$$\frac{\partial f}{\partial t}+v\cdot \nabla f=\nabla_{p}\cdot D\cdot \nabla_{p}f=\frac{\partial F_{\mu }}{\partial \mu }+\frac{1}{p^{2}}\frac{\partial }{\partial p}p^{2}F_{p},$$ (8)

where the components of the diffusive flux vector are

$$F_{\mu }=D_{\mu p}\frac{\partial f}{\partial p}+D_{\mu \mu }\frac{\partial f}{\partial \mu },$$ (9)

$$F_{p}=D_{pp}\frac{\partial f}{\partial p}+D_{p\mu }\frac{\partial f}{\partial \mu }.$$ (10)

Explicit expressions for the components of the diffusion tensor **D** valid for gyroresonant interaction with parallel propagating Alfven waves are given, for example, in Ref. 32. These expressions show that *D_μμ_* is given by Eq. (3) up to a factor of order unity, while *D_pμ_* = *D_μp_* is of order *v_A_*/*c* relative to *D_μμ_* and *D_pp_* is of order (*v_A_*/*c*)^2^. This reflects the ordering of *E*_1_ relative to *B*_1_ mentioned in Eq. (3); $\left|E_{1}/B_{1}|∼O(v_{A}/c\right)$.

In Sec. IV, we will obtain fluid dynamical expressions for the rates at which the cosmic rays and thermal gas exchange momentum and energy collisionlessly. For this purpose, it will be useful to have more general expressions for these quantities from the Fokker-Planck equation. Multiplying Eq. (8) by the parallel momentum *pμ* and integrating over momentum space yields for the rate of parallel momentum exchange

$${\frac{d}{dt}}_{{|}_{wp}}\int p\mu \text{fdpd}\mu \equiv \int p\mu \nabla_{p}\cdot D\cdot \nabla_{p}fp^{2}\text{dpdu}=-\int \left(pF_{\mu }+\mu F_{p}\right)p^{2}dpd\mu ,$$ (11)

where *wp* denotes wave-particle interactions, and in the last step, we have integrated once by parts. From Eqs. (10) and (9), *F_p_* is $O(v_{A}/c)$ relative to *F_μ_*, the first term of the integrand dominates the second. Likewise, multiplying Eq. (8) by particle energy *ϵ* and integrating over momentum space yields for the rate of energy exchange

$${\frac{d}{dt}}_{{|}_{wp}}\int \epsilon \text{fdpd}\mu \equiv \int \epsilon \nabla_{p}\cdot D\cdot \nabla_{p}fp^{2}\text{dpdu}=-\int vF_{p}p^{2}dpd\mu .$$ (12)

The scattering timescale of a few years inferred from the near isotropy and long confinement time of cosmic rays is much shorter than, e.g., the galactic evolution timescale of ∼10^8^ year. This motivates us to solve Eq. (8) approximately, under the assumption of frequent scattering. This is done in Refs. 6, 10, 32, and 33 so we only sketch the derivation here. Equation (8) is ordered based on three timescales: the scattering time *ν*^−1^ is shortest, the light travel time across the cosmic ray density gradient is intermediate, and the global evolution time is the longest. To lowest order *D_μμ_* dominates with nothing to balance it, so *f* is isotropic. Anisotropy appears to next order, such that

$$vn\cdot \nabla f=\frac{\partial F_{\mu }}{\partial \mu },$$ (13)

where $\hat{n}$ is the unit vector along the background magnetic field. The time and perpendicular derivative on the left hand side of Eq. (8) have been ordered out due to the assumptions of long global evolution time and averaging out the fast gyromotion. Integrating Eq. (13) from *μ* to 1 yields

$$F_{\mu }=-v\frac{\left(1-\mu^{2}\right)}{2}n\cdot \nabla f$$ (14)

which upon rearrangement using Eq. (9) yields the momentum space anisotropy

$$\frac{\partial f}{\partial \mu }=-v\frac{\left(1-\mu^{2}\right)}{2D_{\mu \mu }}n\cdot \nabla f-\frac{D_{\mu p}}{D_{\mu \mu }}\frac{\partial f}{\partial p}.$$ (15)

The same ordering is used in Ref. 6, in which the analysis explicitly allows for an inhomogeneous background magnetic field and thermal gas flow. The scale of variation is, however, assumed to be much larger than the wavelengths of gyroresonant Alfvén waves, so the usual Alfvén wave dispersion relation holds. In Ref. 6, the Fokker-Planck equation is first derived in the frame of the waves either co- (“+”) or counter- (“–”) propagating with the cosmic ray drift. In such a frame, the wave is static and scattering conserves energy. The Fokker-Planck equations valid in the two separate frames are then combined and the pitch angle scattering coefficient written in terms of the scattering rates by both species of waves

$$D_{\mu \mu }=D_{\mu \mu }^{+}+D_{\mu \mu }^{-}=\frac{\nu_{+}(1-\mu^{2})}{2}+\frac{\nu_{-}(1-\mu^{2})}{2}.$$ (16)

It is useful to define the composite velocity

$$w\equiv \frac{\nu_{+}w_{+}+\nu_{-}w_{-}}{\nu_{+}+\nu_{-}},$$ (17)

which is the collision frequency weighted mean velocity of the waves that travel at velocities **w**_±_. Since from Eq. (3) the *ν*_±_ are proportional to the intensities of the resonant waves, **w** defines the mean velocity of the wave frame. In a thermal fluid with velocity **V**_*g*_, **w**_±_ = **V**_*g*_ ± *v_A_***n**. Based on these definitions, (15) can then be written as

$$\frac{\partial f}{\partial \mu }=-\frac{vn\cdot \nabla f}{\nu_{+}+\nu_{-}}-w\cdot n\frac{p}{v}\frac{\partial f}{\partial p}.$$ (18)

Using Eq. (17), making the frequent scattering approximation, making the ultrarelativistic approximation *v*/*c* ∼ 1 and dropping some higher order terms in *v_A_*/*c*, the *μ*-averaged Fokker-Planck equation for $\bar{f}$, the isotropic part of *f*, is found to be^6^

$$\frac{\partial \bar{f}}{\partial t}+\frac{\partial }{\partial p^{3}}\left(p^{3}W\right)\cdot \nabla \bar{f}-\left(\nabla \cdot W\right)p^{3}\frac{\partial \bar{f}}{\partial p^{3}}=\nabla \cdot \left(nn\kappa_{∥}\cdot \nabla \bar{f}\right)+\frac{1}{p^{2}}\frac{\partial }{\partial p}p^{2}\kappa_{F}\frac{\partial \bar{f}}{\partial p},$$ (19)

where

$$W\equiv \left\langle \frac{3}{2}(1-\mu^{2})w\right\rangle ,$$ (20)

$$\kappa_{∥}\equiv \frac{v^{2}}{2}\left\langle \frac{\left(1-\mu^{2}\right)}{\nu_{+}+\nu_{-}}\right\rangle ,$$ (21)

and

$$\kappa_{F}\equiv \frac{p^{2}v_{A}^{2}}{v^{2}}\left\langle \frac{2(1-\mu^{2})\nu_{+}\nu_{-}}{\nu_{+}+\nu_{-}}\right\rangle$$ (22)

are the angle averaged transport speed, spatial diffusivity along the background field, and rate of second order Fermi acceleration, respectively. The angle brackets denote averages over *μ*.

In CCRH, which is based on self-confinement, only waves co-streaming with the cosmic rays are present, so only one *ν* is nonzero, $W=W_{sc}=V_{g}\pm \hat{n}v_{A}$, and there is no Fermi acceleration. If balanced extrinsic turbulence is present, *ν*_+_ = *ν*_–_, **W** reduces to **V**_*g*_, and energy flows from waves to particles by the Fermi mechanism.

Although Eq. (19) only accounts for wave-particle interactions, it can easily be generalized to account for radiative and/or collisional processes, which must be included in a complete theory of cosmic ray transport. For the present, however, our interest is in deriving a fluid theory for cosmic rays, so we ignore collisional and radiative terms and proceed with deriving fluid equations by taking moments of Eq. (19).

## IV. FLUID THEORY

### A. From Fokker-Planck to fluid

We are primarily interested in magnetically mediated momentum and energy exchange between the cosmic rays and the thermal fluid. We first consider momentum exchange perpendicular to **B**. Because cosmic ray inertia is negligible, the condition of transverse equilibrium is

$$\nabla_{⊥}P_{c}=\frac{J_{c}\times B}{c},$$ (23)

where *P_c_*, the cosmic ray pressure

$$P_{c}\equiv \frac{1}{3}\int pv\bar{f}p^{2}dp$$ (24)

is the usual kinetic pressure for an isotropic gas and **J**_*c*_ is the cosmic ray current density. The Lorentz force on thermal gas, **J**_*g*_× **B**/*c*, can then be written using Eq. (23) as $J\times B/c-\nabla_{⊥}P_{c}$, while **J**, the total current, is related to **B** by Ampere's Law. Thus, the cosmic ray pressure gradient contributes to the perpendicular force on the thermal gas, and in fact provides 20%–30% of the pressure that supports the gas perpendicular to the galactic plane in the Milky Way and similar galaxies.^34,35^

For momentum exchange parallel to **B**, we use Eqs. (11), (14), and (24). Dropping *μF_p_* relative to *pF_μ_* in Eq. (11), the rate of momentum transfer between the cosmic rays and the waves is then found to be $-n\cdot \nabla P_{c}$. If the waves are in a steady state, momentum transferred to the waves must be transferred in turn to the thermal gas. Thus, cosmic ray momentum exchange with the thermal gas takes the form of a pressure gradient in all directions.

It is possible to include viscous stresses in cosmic ray momentum balance. This was done from a model based on elastic scattering by hard spheres,^36^ and from a model based on pitch angle scattering with no net energy or momentum transfer in Refs. 37 and 38. The hard sphere result was applied to calculating viscous heating of cosmic rays by a shear flow in Ref. 39.

The cosmic ray energy equation follows from multiplying Eq. (19) by particle energy *ϵ* and integrating over momentum. The result is

$$\frac{\partial U_{c}}{\partial t}+\nabla \cdot \left(W_{U}U_{c}\right)+P_{c}\nabla \cdot W_{P}=\nabla \cdot \kappa \cdot \nabla U_{c}+\frac{U_{c}}{\tau_{F}},$$ (25)

where

$$U_{c}\equiv \int p^{2}\epsilon \bar{f}dp$$ (26)

is the energy density in cosmic rays,

$$\kappa \equiv nnU_{c}^{-1}\int p^{2}\epsilon {\bar{\kappa }}_{∥}fdp$$ (27)

is the energy weighted parallel diffusion coefficient,

$$W_{U}=U_{c}^{-1}\int p^{2}\frac{\partial }{\partial p^{3}}(p^{3}w)\epsilon \bar{f}dp,$$ (28)

and

$$W_{P}=P_{c}^{-1}\frac{1}{3}\int p^{3}vw\bar{f}dp.$$ (29)

If $\kappa_{∥}$ depends on energy (as can be inferred from the decrease in confinement time with increasing energy), κ differs from the average diffusion coefficient weighted by the distribution function *f* alone. Because the energy dependence is weak (typically estimated as *ϵ*^0.3–0.6^;^17^), κ is not too different from $\kappa_{∥}$ for particles of the mean energy.

Likewise, **W**_*U*_ and **W**_*P*_ are not the same. In the ultrarelativistic approximation *v* ∼ *c*, *ϵ* ∼ *cp*, the two coincide if **w** is independent of *p*. This is the case of CCRH and for balanced extrinsic turbulence, but it does not hold if, for example, there is a transition from self-confinement to confinement by balanced extrinsic turbulence above some energy. However, due to the power law behavior of $\bar{f}$, which weights all the means toward lower *p*, we will ignore this complication and set $W_{U}=W_{P}\equiv W$. We then rewrite Eq. (25) so as to resemble as closely as possible the energy equation for a thermal gas

$$\frac{\partial U_{c}}{\partial t}+\nabla \cdot \left[W\left(U_{c}+P_{c}\right)\right]=+\nabla \cdot \kappa \cdot \nabla U_{c}+\frac{U_{c}}{\tau_{F}}+W\cdot \nabla P_{c}.$$ (30)

The first term on the right hand side of Eq. (30) represents diffusive transport of cosmic ray energy density. The second term on the right hand side represents Fermi acceleration. If we multiply the last term on the right hand side of Eq. (19) by *p*^2^*ϵdp*, integrate twice by parts, and use Eq. (22), the result is

$$\frac{U_{c}}{\tau_{F}}\equiv \int \frac{\partial }{\partial p}\left(\frac{2p^{4}v_{A}^{2}}{v}\left\langle (1-\mu^{2})\frac{2\nu_{+}\nu_{-}}{\nu_{+}+\nu_{-}}\right\rangle \right)fdp.$$ (31)

We can use Eq. (31) to evaluate the Fermi acceleration timescale *τ_F_* for energization of relativistic particles with *v* ∼ *c* by balanced turbulence with *ν*_±_ = *ν*/2. The result is

$$\tau_{F}^{-1}=\frac{4}{3}\frac{v_{A}^{2}}{c^{2}}\nu ,$$ (32)

which corresponds to a few 10^8^ yr for GeV cosmic Milky Way parameters. This is about an order of magnitude longer than the confinement time, confirming that second order Fermi acceleration by gyroresonant Alfvén waves is not very important under Milky Way conditions. We will drop this term for the remainder of the paper.

We can gain additional insight into the advection and diffusion terms by returning to the second equality in Eq. (12). Using Eqs. (4) and (5) allows us to write the energy equation in the form

$$\frac{\partial U_{c}}{\partial t}+\nabla \cdot F_{U}=-2\int d\omega dk\Gamma_{c}(\omega ,k)I(\omega ,k),$$ (33)

where

$$F_{U}\equiv \int p^{2}v\text{ϵfdpd}\mu$$ (34)

is the cosmic ray energy flux, Γ_*c*_ is defined in Eq. (4), and *I*(*ω*, *k*) is the wave magnetic field power spectrum. Equation (33) shows that energy flows from cosmic rays to growing waves and from damped waves to cosmic rays. This result can be extended to oblique hydromagnetic waves and to fast modes as well as Alfvén modes (Refs. 10 and 40). Evaluating Eq. (34) in the frequent scattering approximation using Eqs. (9) and (14) leads to

$$F_{U}=W\left(U_{c}+P_{c}\right)-\kappa \cdot \nabla U_{c},$$ (35)

which is consistent with Eq. (30).

There are two ways to proceed in evaluating the wave-particle interaction term on the right hand side of Eq. (33), and they lead to equivalent results. One is to rewrite Γ_*c*_ by replacing *∂f*/*∂μ* in A, defined in Eq. (5), by its value in the frequent scattering approximation, Eq. (18) evaluated in the rest frame of the fluid. Keeping only the dominant terms in *v*/*v_A_*, the result is

$$A_{\pm }=\frac{2\nu_{\mp }}{\nu_{+}+\nu_{-}}\frac{\partial f}{\partial p}\mp \frac{v^{2}n\cdot \nabla f}{v_{A}p(\nu_{+}+\nu_{-})}.$$ (36)

If we substitute Eq. (36) into Eq. (4), use the resulting expression in Eq. (33) together with the fact that the energy density in Alfven waves is twice the magnetic energy in the wave, and transform back from the rest frame of the fluid to the frame of a stationary observer, we arrive at

$$\frac{\partial U_{c}}{\partial t}+\nabla \cdot F_{U}=W\cdot \nabla P_{c}.$$ (37)

Alternatively, we can use the expressions for the components of the diffusion tensor **D** and corresponding approximation to the anisotropy derived in Ref. 32 to substitute for *F_p_* in Eq. (25). Either way, we interpret the right hand side of Eq. (30) as the sum of work done on the thermal gas by the cosmic ray pressure gradient ($V_{g}\cdot \nabla P_{c}$) and energy exchanged with the waves ($(W-V_{g})\cdot \nabla P_{c}$). The rate of energy transfer to waves is scaled by *v_A_* and is weighted by the relative intensities of waves co-and counterpropagating relative to the cosmic ray anisotropy; it is largest for self-confinement and zero for confinement by balanced extrinsic turbulence.

There is a consistency condition for this energy transfer picture to hold: $A_{+}$ must be positive. This can be expressed as a condition on the cosmic ray spatial gradient

$$\frac{L}{\lambda }\equiv \frac{f}{\left|\nabla_{∥}f\right|}\frac{\nu_{+}+\nu_{-}}{c}<\frac{L_{max}}{\lambda }=\frac{c}{2v_{A}}\frac{\nu_{+}+\nu_{-}}{\nu_{-}}\frac{f}{\left|df/d\ln p\right|}.$$ (38)

Although Eq. (38) implies that the streaming instability turns off only if extrinsic turbulence is present, it can also happen if there is no excitation or damping of waves other than the cosmic rays themselves, as shown in Figure 2.

### B. Equations for the waves

In order to close the system of fluid equations, we need equations for the amplitudes of the waves. An equation for wave energy valid for CCRH was given in Ref. 15. It combines advection by the thermal background flow, changes in amplitude with changes in properties of the background according to the WKB approximation, and local excitation and damping

$$\frac{\partial }{\partial t}\frac{\delta B^{2}}{4\pi }=-\nabla \cdot F_{w}+u\cdot \nabla \frac{\delta B^{2}}{8\pi }-v_{A}n\cdot \nabla P_{c}-L.$$ (39)

In Eq. (39), *δB*^2^/4*π* is the wave magnetic and kinetic energy

$$F_{w}\equiv \frac{\delta B^{2}}{4\pi }\left(nv_{A}+\frac{3}{2}u\right)$$ (40)

is the wave energy flux, $-v_{A}n\cdot \nabla P_{c}$ is the rate of energy per volume transferred to the waves by the cosmic rays by the streaming instability, and *L* represents non-cosmic ray sources and sinks of wave energy. Other than the excitation and damping terms, Eq. (39) is fully consistent with Refs. 41 and 42. The waves exert a force on the thermal fluid, which was also calculated in Ref. 41 and is included in Sec. IV E.

Equation (39) has rarely been solved in full. Recall that under Milky Way conditions, *δB*^2^/*B*^2^ ∼ 10^−6^, while 8*πP_c_*/*B*^2^ ∼ 1. This implies that $\left|n\cdot \nabla P_{c}|/|\nabla \cdot F_{w}\right|$ is $O(10^{6})$. Therefore, $nv_{A}\cdot \nabla P_{cr}$ is most likely balanced by *L*. This local approximation to the wave energy density generally allows a straightforward solution for the wave energy which is discussed in more detail below. For an example in which the steady state version of Eq. (39) is solved including wave transport and WKB effects, see Ref. 43.

We now generalize Eq. (39) to allow for waves traveling in both directions. We define two wave energy flux vectors

$$F_{w\pm }\equiv \frac{\delta B_{\pm }^{2}}{4\pi }\left(\pm nv_{A}+\frac{3}{2}V_{g}\right),$$ (41)

and write two wave energy evolution equations

$$\frac{\partial }{\partial t}\frac{\delta B_{\pm }^{2}}{4\pi }=-\nabla \cdot F_{w\pm }+V_{g}\cdot \nabla \frac{\delta B_{\pm }^{2}}{8\pi }\mp \frac{\nu_{\pm }}{\nu_{+}+\nu_{-}}nv_{A}\times \nabla P_{c}-G_{\pm }+S_{\pm }.$$ (42)

The *G*_±_ and *S*_±_ in Eq. (42) represent damping and non-cosmic ray sources of Alfven waves, respectively. They are related to the *L* introduced in Eq. (39) by $G_{+}+G_{-}-S_{+}-S_{-}=L$. Damping mechanisms are described in Ref. 19, so we merely summarize the most importance mechanisms here. In weakly ionized gas, the waves propagate primarily in the plasma component and are damped by collisions between ions and neutrals. This is usually so effective that gyroresonant waves are essentially wiped out, decoupling all but the transverse pressure gradient aspect of cosmic ray coupling to the thermal gas and likely invalidating the diffusion approximation. In hot, fully ionized gas, nonlinear Landau damping, which occurs when thermal ions interact resonantly with the pressure gradient force associated with Alfven wave packets, is an efficient process. A third mechanism, sometimes called turbulent damping, is due to the shearing apart of Alfven waves propagating through a turbulent background magnetic field. The turbulent damping rate has not yet been calculated rigorously, only approximately, and a better understanding of how it operates and how it affects the streaming instability is needed. In Sec. VI, we will discuss the effect of ion Landau damping on the confinement of cosmic rays in turbulent, high *β* plasmas.

Other than cosmic rays, a turbulent cascade is the most likely source of gyroresonant waves. Although the power input to the turbulent cascade is about 2 orders of magnitude larger than the mean cosmic ray power input, the decrease in fluctuation amplitude along the cascade suggests that the cosmic rays are a stronger source at the gyroscale itself. Moreover, the MHD cascade is thought to be anisotropic, such that the gyroresonant waves have $k_{⊥}/k_{∥}\gg 1$ and thus scatter cosmic rays inefficiently.^30,44^ The waves in the cascade that are most important for scattering are fast magnetosonic waves, which are generated by compressibility effects and represent a small part of the turbulent energy spectrum. Furthermore, the sources of interstellar turbulence are most intense at the galactic midplane, so the turbulence is likely not globally balanced. Thus, **w** may be close to **n***v_A_* and points down the cosmic ray pressure gradient even if the waves are not self-excited. Cosmic rays will be transported relative to the gas at close to the Alfven speed. However, whether to call the resultant heating turbulent heating or cosmic ray heating should be decided based on the problem at hand.

### C. A self-consistent model

It is instructive to make a steady state model of cosmic ray transport by solving Eq. (30) with the velocity **W** and diffusion tensor κ calculated from Eq. (42) in the local approximation, adding a term that drives waves equally in both directions. For simplicity we assume that $n=\hat{z}$, *v_A_* is constant, *P_c_* depends on *z* only, and the waves are linearly damped such that $G_{\pm }=\Gamma_{d}\delta B_{\pm }^{2}/4\pi$. Equations (42) then become

$$0=\mp \frac{E_{\pm }}{E}v_{A}\frac{dP_{c}}{dz}-\Gamma E_{\pm }+2\Gamma E_{0},$$ (43)

where *E*_±_ are the wave energy densities in the co and counterpropagating directions, *E* is their sum, and 2Γ*E*_0_ represents driving such that in the absence of cosmic rays, $E_{+}=E_{-}=E_{0}/2$. In writing Eq. (43), we have used the proportionality of *ν*_±_ to *E*_±_ (see Eq. (3)).

It is convenient to introduce a dimensionless length *ζ* ≡ *z*/*L*, a dimensionless cosmic ray pressure *ψ* ≡ *P_c_*/*P_c_*_0_, and an Alfven travel time *τ_A_* ≡ *L*/*v_A_*. We can then solve for *E*_±_ from

$$\frac{E}{E_{0}}=1+{\left[1+\Xi^{2}{\left(\frac{d\psi }{d\zeta }\right)}^{2}\right]}^{1/2},$$ (44)

$$\frac{E_{+}-E_{-}}{E_{+}+E_{-}}=\frac{W}{v_{A}}=-\frac{\Xi \frac{d\psi }{d\zeta }}{1+{\left[1+\Xi^{2}{\left(\frac{d\psi }{d\zeta }\right)}^{2}\right]}^{1/2}},$$ (45)

where

$$\Xi \equiv \frac{P_{c0}}{E_{0}\Gamma \tau_{A}}$$ (46)

measures the strength of cosmic ray driving and damping relative to the other driving and damping processes that determine the amplitudes of the waves. Large Ξ corresponds to strong cosmic ray driving; *P_c_*_0_/*τ_A_* ≫ Γ*E*_0_.

Equations (45) and (46) when used in Eq. (30) fully determine steady state cosmic ray transport in this 1D case. With the further definitions *κ*_0_ ≡ *κE*/*E*_0_, the introduction of a parameter Λ that measures the relative important of advection and diffusion

$$\Lambda \equiv \frac{Lv_{A}}{\kappa_{0}},$$ (47)

and some algebraic simplifications, Eq. (30) takes the form

$$\left(4\Xi \psi +\Lambda^{-1}\right)\frac{d^{2}\psi }{d\zeta^{2}}+3\Xi {\left[1+\Xi^{2}{\left(\frac{d\psi }{d\zeta }\right)}^{2}\right]}^{1/2}{\left(\frac{d\psi }{d\zeta }\right)}^{2}=0.$$ (48)

Equation (48) has some interesting features. If we take the limit of large Ξ and adopt the convention *dψ*/*dζ* < 0, it reduces to

$$\frac{d}{d\zeta }\left(4\psi +\frac{1}{\Xi \Lambda }\right)=\frac{d\psi }{d\zeta },$$ (49)

which is first order instead of second order. Apart from the differences in notation, Eq. (49) is the fluid version of the kinetic transport equation derived in Ref. 33 under the assumption that the cosmic ray anisotropy is set by balancing wave growth with wave damping. The reduction of order comes about because requiring the waves be marginally stable adds a constraint. This concept has been extended in some treatments; see Sec. IV D.

The case of small Ξ is also of interest. In this case Eq. (48) reduces to

$$\frac{d^{2}\psi }{d\zeta^{2}}=0,$$ (50)

the turbulence is balanced, and cosmic ray propagation is purely diffusive.

**FIG. 1.**
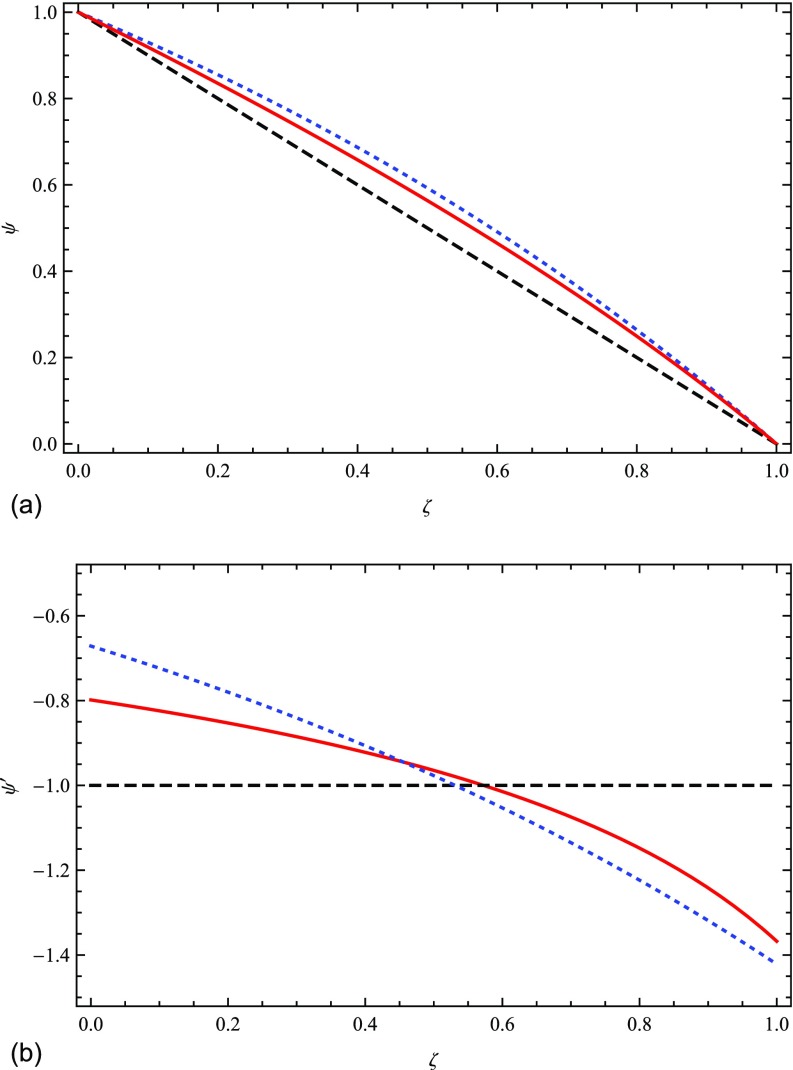
Left panel: Comparison of the scaled cosmic ray pressure *vs* scaled distance for 3 different transport models. The black dashed line is the diffusion dominated limit. The red solid line is the solution of Eq. (48) for Λ = 1, Ξ = 0.25. The blue dotted line is the solution of Eq. (30) nondimensionalized in the same way as Eq. (48) with *WL*/*κ* = 0.75. Right panel: Comparison of the scaled pressure gradients in the 3 cases.

Figure 1 illustrates the effect of the transport model on *P_c_* and $\nabla P_{c}$ for three different cases with the same boundary conditions: $\psi (0)\equiv P_{c}(0)/P_{c0}=1,\;\psi (1)=0$. The red curves correspond to the self-consistent model with Ξ = 0.25 and Λ = 1, the black curves to Ξ = 0 (diffusion dominates), and the blue curves to a solution of Eq. (30) in which the relative values of the streaming and advection times are the same as the self-consistent model at *ζ* = 0. While the pressure distributions are quite similar, the pressure gradients are rather different, demonstrating that the dynamical effects of cosmic rays depend on the transport model.

### D. Empirical treatments

Galaxies are complex systems, and it is not easy to fully account for magnetic fields and cosmic rays in galactic structure and evolution. Here, we mention some approximate treatments that have appeared and put them in the context of GCRH.

First and most simply, it is possible to include the dynamical effect of cosmic ray pressure without including a global magnetic field by assuming extrinsic turbulence. Cosmic ray heating must of course be omitted. Galactic winds and cosmic ray feedback are modeled in Ref. 45 using this approach. Diffusion can be included without modeling either the large scale magnetic field or its turbulent component by assuming that it is due, at least in part, to magnetic field line random wandering through space (we know that this cannot be the full picture in the Milky Way, however, due to the energy dependence of the cosmic ray lifetime; see Sec. II). However, accounting for unresolved magnetic structure provides some freedom in choosing κ.

Ignoring field line wandering for the moment, in Sec. IV C, we showed how *κ* is determined self consistently by the balance between wave driving and damping. Equation (49) therefore suggests another variant on the model: introduce a super-Alfvenic transport velocity and subsume the diffusive flux in Eq. (30) into the streaming flux, setting *κ* ≡ 0 and replacing *v_A_* by *f_s_v_A_*, *f_s_* > 1 in the energy flux

$$v_{A}n(U_{c}+P_{c})-\kappa nn\cdot \nabla U_{c}\to f_{s}v_{A}n(U_{c}+P_{c}).$$ (51)

This corresponds to self-confinement—the waves are generated by the cosmic rays themselves—but due to some external wave dissipation process(es), the cosmic ray anisotropy must exceed the threshold for the streaming instability [A>0 in Eq. (5) or *v_D_*/*v_A_* > 1 in Eq. (6)]. This approach was taken by Refs. 46 and 47. However, the plasma heating term remains $v_{A}n\cdot \nabla P_{c}$.

Finally, Ref. 48 implemented cosmic ray transport, including heating, with the sound speed *v_S_* substituted for *v_A_*. This provides a way to explore the effects of cosmic ray transport, including streaming vs diffusion,^49^ but there is no clear plasma physics basis for it.

### E. Summary of fluid equations

We now summarize the fluid equations for thermal gas, cosmic rays, and waves, using subscripts *g*, *c*, and *w* to denote these quantities. The continuity equation for the thermal gas is

$$\frac{\partial \rho_{g}}{\partial t}=-\nabla \cdot \left(\rho_{g}V_{g}\right).$$ (52)

The momentum equation includes cosmic ray and wave stresses

$$\frac{\partial \rho V_{g}}{\partial t}+\nabla \cdot \left(\rho_{g}V_{g}V_{g}\right)=-\nabla \left(P_{g}+P_{c}+\frac{\delta B^{2}}{8\pi }\right)+\frac{\left(\nabla \times B\right)\times B}{4\pi }-\rho_{g}\nabla \Phi ,$$ (53)

where Φ is the gravitational potential, which is determined from Poisson's equation including all sources. Equation (53) can be generalized to include viscous stresses, radiation pressure, anisotropic gas pressure, etc.

The energy equation for the thermal gas is

$$\frac{\partial U_{g}}{\partial t}+\nabla \cdot \left(V_{g}U_{g}\right)=\rho_{g}\frac{dQ}{dt}-P_{g}\nabla \cdot V_{g},$$ (54)

where *U_g_* is the thermal energy density and all nonadiabatic effects, in particular, the wave dissipation terms *G* appearing in Eq. (42) are accounted for in *ρ_g_dQ*/*dt*. Other nonadiabatic terms that are often important in astrophysics include radiative heating and cooling and thermal conduction.

The energy equation for cosmic rays is Eq. (30), rearranged and with Fermi acceleration dropped

$$\frac{\partial U_{c}}{\partial t}+\nabla \cdot \left[W\left(U_{c}+P_{c}\right)-\kappa \cdot \nabla U_{c}\right]=W\cdot \nabla P_{c},$$ (55)

where **W** is given by Eq. (20) [which follows from Eq. (17)], and κ by Eq. (27). The behavior of the waves is given by Eq. (42), which can be combined into

$$\frac{\partial }{\partial t}\frac{\delta B^{2}}{4\pi }=-\nabla \cdot F_{w}+V_{g}\cdot \nabla \frac{\delta B^{2}}{4\pi }-(W-V_{g})\cdot \nabla P_{c}-G+S,$$ (56)

where $G=G_{+}+G_{-},\;S=S_{+}+S_{-}$, and

$$F_{w}\equiv \left(W+\frac{1}{2}V_{g}\right)\frac{\delta B^{2}}{4\pi }.$$ (57)

However, Eq. (42) must be solved separately to calculate **W** self consistently.

The background magnetic field obeys the usual ideal MHD induction equation

$$\frac{\partial B}{\partial t}=\nabla \times \left(V_{g}\times B\right).$$ (58)

The system of Equations (52)–(58) obeys the energy conservation equation

$$\frac{\partial U}{\partial t}=-\nabla \cdot F+\rho_{g}\frac{dQ}{dt}-G+S,$$ (59)

where

$$U\equiv \frac{1}{2}\rho_{g}V_{g}^{2}+U_{g}+U_{c}+\frac{\delta B^{2}}{4\pi }+\frac{B^{2}}{8\pi }+\rho \Phi$$ (60)

is the total energy density and

$$F\equiv V_{g}\left(\frac{1}{2}\rho V_{g}^{2}+U_{g}+P_{g}+\rho \Phi \right)-\frac{(V_{g}\times B)\times B}{4\pi }+W(U_{c}+P_{c})-\kappa \cdot \nabla U_{c}+F_{w}$$ (61)

is the total energy flux.

We see from Eq. (59) that if we identify *G* as a component of *ρ_g_dQ*/*dt*, then those terms disappear from the energy equation: dissipated wave energy goes into heating the gas. The *S* term represents turbulent driving and is not fully addressed by this model; for example, we have not include a turbulent Reynolds stress in Eq. (53). However, if we assume that *S* represents driving of a turbulent cascade that is dissipated at small scales, that we can assume that *S* as well as *G* is partially cancelled by *ρ_g_dQ*/*dt*.

**FIG. 2.**
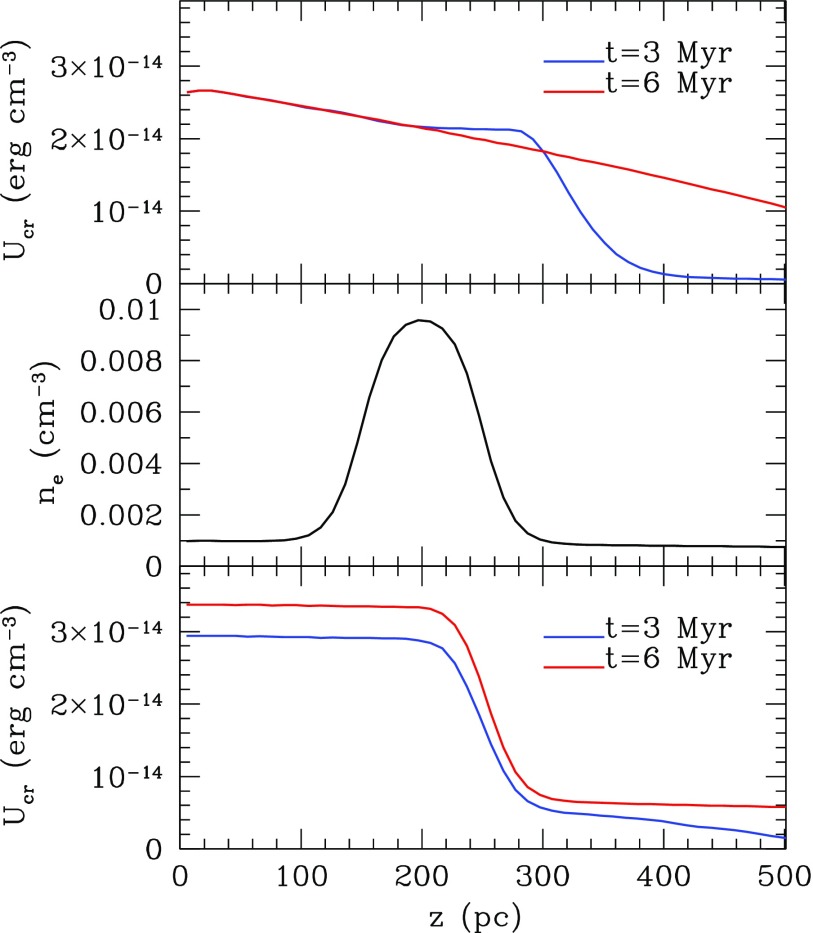
Top panel: Cosmic ray energy density at 3 and 6 Myr after a source is turned on at the left boundary and cosmic rays stream into a plasma with a constant background magnetic field and negative density gradient. Behind a front, which travels outward at the Alfven speed, *U_c_* converges to a steady solution with $U_{c}\rho_{g}^{-\gamma_{c}/2}$ constant. The middle and bottom panels pertain to a different numerical experiment in which a cloud of denser plasma, with the density profile shown in the middle panel, is placed some distance from the boundary. This leads to a “bottleneck” between the density maximum and the source within which there are no waves and *U_c_* is constant. Wave locking begins beyond the density maximum and the cosmic rays follow *ρ_g_* as in the top panel. Based on J. L. Wiener *et al.*, Mon. Not. R. Astronom. Soc. **467**, 646 (2017).

The self-confined, wave locked (*κ* = 0) case leads to interesting steady state behavior that was predicted in Ref. 33 and only recently verified through numerical simulation.^50^ Suppose **W **=** n***v_A_* and write *P_c_* = (*γ_c_* – 1)*U_c_*. Then, it follows from Eq. (55) that $U_{c}v_{A}^{\gamma_{c}}$ is constant. If *v_A_* decreases in the direction of propagation then *U_c_* must increase. But we know from Eq. (36) that Alfven waves are only excited if *U_cr_* is *decreasing*. It was argued in Ref. 33 that the only equilibrium solution is one in which *U_cr_* is flat, there are no Alfven waves, and the streaming velocity is the minimum value of *v_A_*. This is confirmed by the time dependent simulations of Ref. 50 as shown in Figure 2.

## V. APPLICATIONS

### A. Gravitationally stratified gas in galaxies

We consider the equilibrium and stability of the interstellar medium perpendicular to the galactic plane. Because galactic disks are thin, we can treat this as a 1D problem. Taking the magnetic field to be parallel to the galactic plane, the condition for vertical equilibrium is

$$\frac{d}{dz}\left(P_{g}+P_{c}+\frac{B^{2}}{8\pi }\right)=-\rho g_{z}.$$ (62)

**FIG. 3.**
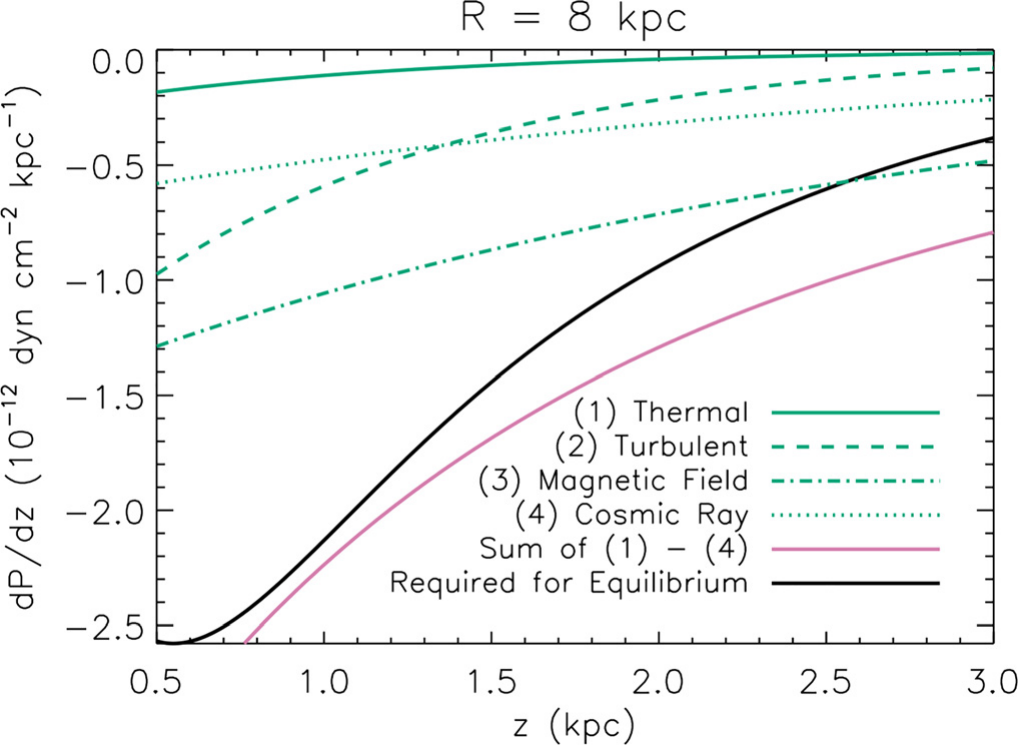
Contributions of the thermal pressure, dynamical pressure *ρv*^2^, cosmic ray pressure, and gas pressure to the vertical equilibrium of ionized gas in the galaxy NGC 891. Because the galaxy is seen edge on, the galactocentric radius of the gas, and hence the strength of vertical gravity, is unknown; here, it is assumed to be 8 kpc. The gas pressure and density are measured spectroscopically; the magnetic and cosmic ray pressures are from measurements of synchrotron radiation. None of these components can support the gas alone, but their sum (red curve) is adequate, and slightly exceeds what is required (black curve). Based on E. Boettcher *et al.*, Astrophys. J. **832**, 118 (2016).

Magnetic fields and cosmic rays play a key role in the equilibrium as thermal and dynamical pressures are generally inadequate to support the gas at its observed height; see Figure 3, which shows the comparison for the galaxy NGC 891.^35^

The stability of systems which satisfy Eq. (62) without cosmic rays was treated in Ref. 51; cosmic rays were later added by Ref. 52 for a specific family of equilibria and generalized by Ref. 53 (2D equilibria were considered in Ref. 54). The general condition for stability in 1D is

$$-\frac{d\rho_{g}}{dz}>\frac{\rho_{g}^{2}g_{z}}{\gamma_{g}P_{g}+\gamma_{c}P_{c}},$$ (63)

where the *γ* factors are defined by the relationships between pressure and density perturbations such that *δP_g_*_,__*c*_/*P_g_*_,__*c*_ = γ_*g,c*_*δρ_g_*/*ρ_g_*. While the background magnetic field *B* does not appear explicitly in Eq. (63), it appears implicitly, through its effect on the stratification. The instability is driven by the energy released when gas falls into the gravitational potential well, but opposed by magnetic tension and by the work done in compressing the gas as it slides along the fieldlines. Equation (63) is derived assuming the cosmic rays are locked to the gas rather than streaming relative to it: a treatment that accounts for streaming, diffusion, and collisionless heating has not, to our knowledge, been carried out.

Paper 52 assessed the stability of a family of models with *g_z_* constant, *P_g_* = *ρ_g_a*^2^ for constant *a*^2^, and constant ratios of pressures $P_{c}/P_{g}\equiv \beta ,\;B^{2}/8\pi P_{g}\equiv \alpha$ (in the notation of Ref. 52). Equation (62) then has exponentially decaying solutions $\rho (z)=\rho (0)e^{-z/H}$, where

$$H=\frac{a^{2}(1+\alpha +\beta )}{g_{z}}$$ (64)

and Eq. (63) gives

$$1+\alpha +\beta <\gamma_{g}+\beta \gamma_{c}$$ (65)

for stability. It was argued that *γ_g_* = 1 because interstellar gas cools efficiently, and, because Ref. 52 predates the theory of cosmic ray coupling parallel to **B**, *γ_c_* was set equal to zero. Equation (65) therefore predicts that the system is always unstable.

**FIG. 4.**
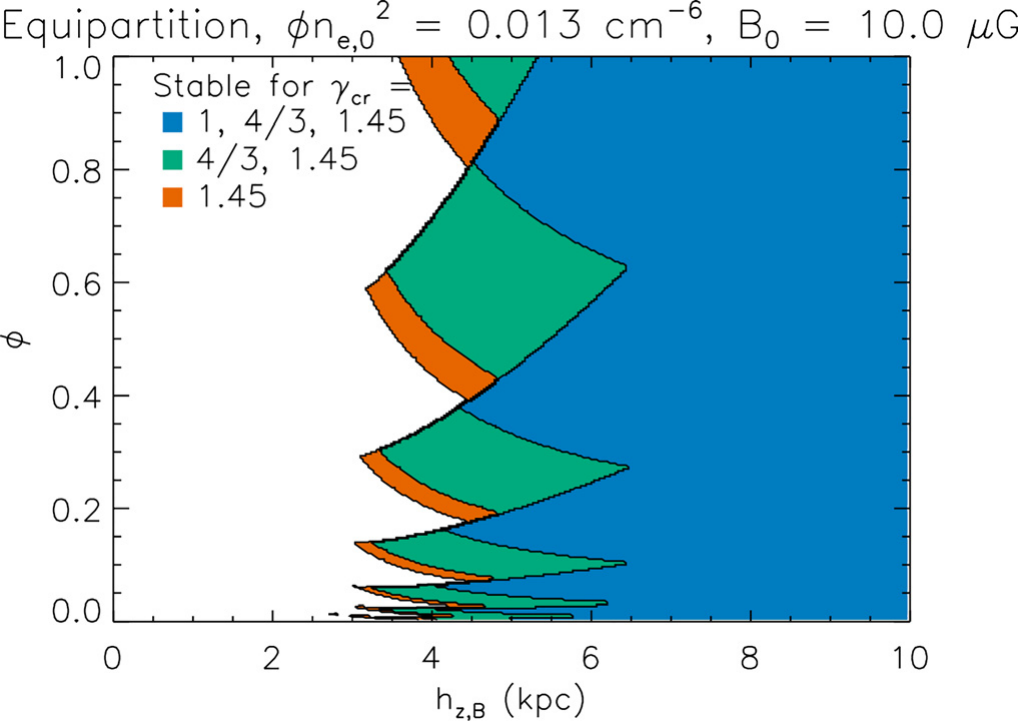
Stability diagram for models of the ionized gas layer in NGC 891. The magnetic field is assumed to vary with height as $B_{0}e^{-z/h_{z,B}}$ where *B*_0_ = 10*μG*. The parameter ϕ measures the clumpiness of the gas such that the mean density is proportional to $\phi^{-1/2}$. If *γ_g_* = 1, there are no stable models for *γ_c_* = 0. As *γ_c_* is increased from 1 (mimicking the effects of diffusion) to 4/3 (the value for an ultrarelativistic gas) to 1.45, which accounts for nonrelativistic cosmic rays, smaller *h_z_*_,__*b*_ values are become stable. The jagged stability boundaries are due to choosing discrete values of the galactocentric radius *R* such that smaller values of ϕ are stable at larger *R*. Based on E. Boettcher *et al.*, Astrophys. J. **832**, 118 (2016).

Figure 4 shows the results of applying Eq. (63) to equilibrium models of NGC 891 of the kind shown in Figure 3 for models with a range of magnetic field scale heights and ionized gas filling factors, which are not well constrained by the observations. If *γ_c_* = 0, *γ_g_* = 1, none of the models are stable. If cosmic ray coupling is assumed, such that *γ_c_* increases from zero to the appropriate value for a semirelativistic gas, a large part of the parameter space is stable. Stabilization is due to the work required to compress the cosmic ray gas.

### B. Galactic winds

The full suite of cosmic ray effects comes into play when we consider the role of cosmic rays in launching and energizing galactic winds. The morphological and spectroscopic evidence for winds is reviewed by Ref. 55; their importance in the evolution of galaxies and the intergalactic medium is increasingly recognized.

**FIG. 5.**
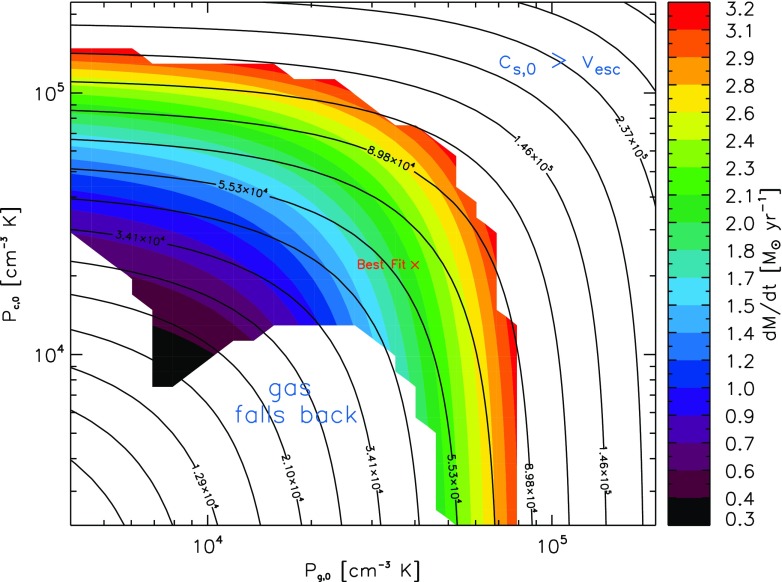
Mass loss rates and flow regimes for hybrid thermally and cosmic ray driven winds parameterized by their base and cosmic ray and thermal pressures. Color indicates mass loss rate in solar masses per year. The contours are curves of constant total base pressure The best joint fit to the soft x-ray and radio synchrotron emission from the inner Milky Way is labeled with a red cross. Gas and cosmic rays contribute about equally to launching this wind, and it could not be launched by thermal pressure alone. From Everett *et al.*, Astrophys. J. **711**, 13 (2010). Copyright 2010 IOP.

CCRH was first applied to galactic winds in Ref. 15 and later used to model the soft x-ray emission^56^ and joint x-ray and synchrotron emission^57^ from the inner Milky Way. These models were based on steady state flow along magnetic flux tubes of prescribed shape. Figure 5 shows how the mass loss rate for a wind from the inner Milky Way varies with cosmic ray and thermal gas pressure. There are three regimes: too little pressure to launch a wind, so high a pressure that the flow is supersonic everywhere [by “supersonic” we refer to the speed at which the flow has a critical point; it differs from the ordinary (*dP*/*dρ*)^1/2^ in accounting for Alfvenic streaming and Alfven wave pressure] and an intermediate regime where the wind is subsonic at the base and transitions to supersonic at a critical point. All models are compared with Milky Way x-ray and continuum observations through a *χ*-squared test and the best fit, indicated by a cross, has roughly equal thermal and cosmic ray base pressures. Within the critical point regime, contours of constant mass flux are similar but not identical to contours of constant pressure, with cosmic ray dominated winds having slightly lower mass fluxes than thermal winds at the same pressure. This occurs because cooling by adiabatic expansion is slower for relativistic particles than for nonrelativistic ones; therefore, cosmic rays deposit their momentum over longer distances. Therefore, a cosmic ray driven wind has a lower mass flux but a higher asymptotic velocity than the equivalent thermal pressure wind.

**FIG. 6.**
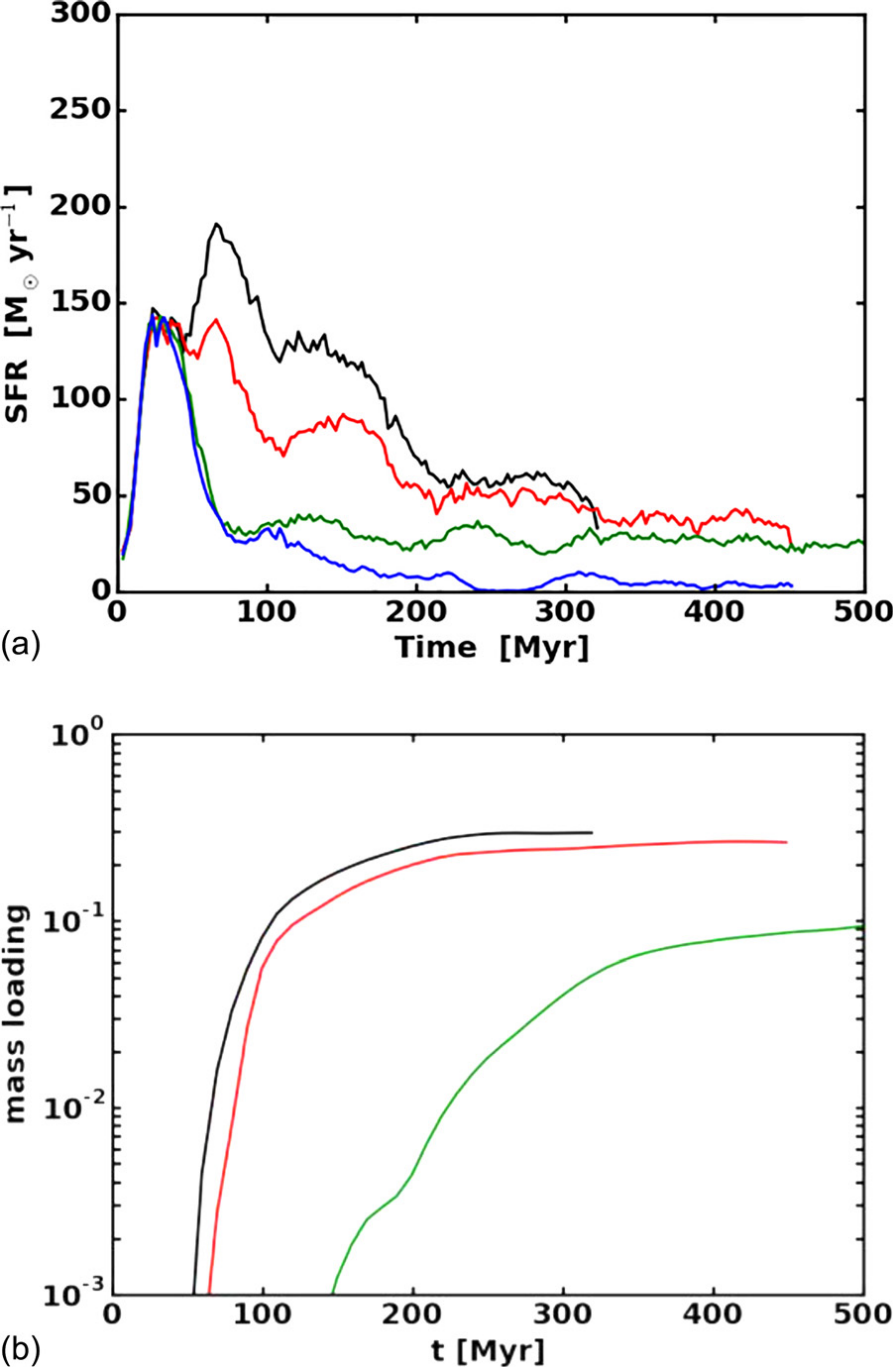
Star formation (top) and mass loss rates (bottom) in numerical simulations of thermally and cosmic ray driven winds for four simply parameterized models of cosmic ray transport. Diffusion is set to zero in all models. Blue curves represent balanced extrinsic turbulence with no transport relative to the gas, green is transport at *v_A_*, and red and black correspond to setting the super = Alfvenic streaming parameter *f_s_* introduced in Eq. (51) to 4 and 8, respectively. Based on M. Ruszkowski *et al.*, Astrophys. J. **834**, 208 (2017).

Steady state winds cannot address the problem of stellar feedback, i.e., how winds driven by energy provided from stars affect the star formation rate itself. Figure 6 shows the rates of star formation and wind mass loss rates in 3D, time dependent, MHD simulations of galaxy formation.^47^ The star formation rate responds to the local gas density and kinematics in a prescribed way, a supernova rate based on the star formation rate is chosen according to widely accepted theories of stellar evolution, and it is assumed that a certain fraction of supernova energy—10% in all cases discussed here—is put into cosmic rays. The treatment of star formation is very standard; see, e.g., Ref. 58.

In all cases, the star formation rate peaks and then declines over time. The drop is largest if there is no cosmic ray transport relative to the gas. In this case, cosmic ray pressure inflates the disk, lowering the mean density and therefore the role of self-gravity and the probability of gravitational collapse necessary to convert gas to stars. However, no wind is driven in this case, because cosmic ray pressure is insufficient to lift the gas out of the gravitational potential well of the galaxy. Whether this cosmic ray pressure supported disk is Parker unstable [see Eq. (63)] cannot be addressed by these rather coarsely resolved simulations. As the transport velocity is increased (increasing *f_s_*), a wind with mass loss rate correlating with *f_s_* is launched. These models also show reduction in the star formation rate, but the suppression varies inversely with *f_s_*. We attribute this to efficient cosmic ray transport out of the densest regions of the disk; cosmic ray pressure is sufficient to drive a low density wind, partially depleting the mass supply, but the reduced cosmic ray pressure is less effective at reducing the mean gas density in the disk.

These results represent the first implementation of cosmic ray streaming in a full MHD simulation. As more physical processes are modeled with greater fidelity, the models will improve. We are left with the conclusion, however, that *cosmic ray transport matters*.

## VI. GALAXY CLUSTERS: COSMIC RAY PROPAGATION AT HIGH *B*

Galaxy clusters are the most massive gravitationally bound entities known in the Universe. The largest contain thousands of galaxies, substantial dark matter in an unknown form, and diffuse, magnetized plasma at temperatures of a keV and more. Rich clusters usually have a central dominant galaxy with supermassive nuclear black hole, which may show signatures of mass accretion such as jets, x-rays, or synchrotron radiation. The source of accreted material is thought to be the intracluster medium (ICM), and the regulation of accretion by heat and momentum deposited in the ICM by nuclear activity is known as *black hole feedback*.

Cosmic ray transport affects three important problems in ICM physics. The first is the origin of the relativistic electrons that produce extended synchrotron halos in some clusters. The Alfven travel time between the cluster core and the halo exceeds the radiative loss time, suggesting that either cosmic ray electron transport is super-Alfvenic, or the electrons originate in the halo of the cluster.^59^ The second is whether collisionless cosmic ray heating contributes significantly to energy balance in the ICM,^60,61^ i.e., whether there is cosmic ray feedback. The third is the nondetection of *γ*-rays from cosmic ray interactions in the cluster cores. The upper limits on such emission are stringent,^62^ suggesting either that the rate of cosmic ray escape from galaxies and AGN into the ICM is much lower than predicted or that cosmic rays are transported very quickly from the cluster core to the lower density plasma at the outskirts.

The ICM has lower collisionality and higher plasma *β* (10–100) than interstellar gas (*β* ∼1), potentially putting cosmic ray propagation and its fluid behavior into a new regime as far as the types of waves that are present and how they damp. These effects may be important in all high *β* astrophysical plasmas.

The dispersion relation of hydromagnetic waves in high *β* plasmas was analyzed asymptotically in Ref. 63. At wavenumbers *k* such that the thermal ion gyroradius $r_{i}=v_{i}/\omega_{ci},\;v_{i}\equiv \sqrt{2k_{B}T_{i}/m_{i}}$ satisfies *kr_i_* ∼ 1, the waves become dispersive. The lowest order corrections yield, for parallel propagating waves

$$\omega ∼kv_{A}\pm \frac{k^{2}v_{i}^{2}}{4\omega_{ci}},$$ (66)

where the ± signs denote right and left circular polarization, respectively. According to Eq. (4), both wave polarizations can be destabilized by streaming anisotropy; since the phase velocity of the left circularly polarized wave is less than *v_A_*, this effect lowers the threshold anisotropy for streaming instability. However, from Eq. (1), only cosmic rays with $\mu <v_{i}^{2}/v_{A}c\ll 1$ will resonate with these modified waves. For the keV temperatures, *μ*G magnetic fields, and number densities of 10^−2^–10^−3^ typical of galaxy cluster cores, relatively few cosmic rays will be affected, and the bulk streaming velocity should not be much retarded.

Parallel propagating waves with *kr_i_* ∼ 1 are thermally cyclotron damped at the rate

$$\Gamma_{cyc}\approx \frac{\sqrt{\pi }}{2}\frac{\omega_{ci}}{kr_{i}}e^{-1/{\left(kr_{i}\right)}^{2}},$$ (67)

which will overwhelm wave growth due to the streaming instability for $kr_{i}>1/\sqrt{\ln(n_{i}/n_{c})}$. Thus, the waves that resonate with cosmic rays having pitch angles in a narrow cone around *π*/2 will be strongly damped. The issue of how such particles scatter was first raised in a general context in Ref. 64 and addressed in Ref. 65, where it was shown that mirroring from the longer wavelength waves excited by cosmic rays with larger *μ* provides adequate scattering.

Landau damping of oblique waves by thermal ions, which affects all waves, is a more serious issue. The damping rate for waves propagating to the background magnetic field at an angle *θ* ≪ 1 and *kr_i_* < *v_A_*/*v_i_* can be approximated as^63^

$$\Gamma_{L}\approx \frac{\sqrt{\pi }}{4}kv_{i}\;{\text{tan}}^{2}\theta .$$ (68)

Up to now we have considered only parallel propagating waves because the growth rate of the streaming instability is maximal at *θ* = 0. But arguably *θ* = 0 is only possible if the background magnetic field is completely uniform. We assume a turbulent MHD cascade is present and follow Ref. 66 in estimating the maximum perpendicular wavelength *λ*_⊥_(*r_c_*) that must accompany a wave with parallel wavelength of order the cosmic ray gyroradius *r_c_*. The result is

$$\theta_{min}\equiv \frac{r_{c}}{\lambda_{⊥}(r_{c})}∼{\left(\frac{r_{c}\epsilon_{d}}{v_{A}^{3}}\right)}^{1/4},$$ (69)

where *ϵ_d_* is the dissipation rate of turbulent energy density, and can be expressed in terms of the scale *L_MHD_* at which the cascade becomes Alfvenic as $\epsilon_{d}∼v_{A}^{3}/L_{MHD}$. In a high *β* plasma, we might expect the turbulent velocity at the outer scale to be superAlfv6enic, in which case *L_MHD_* would be less than the outer or driving scale. Using Eq. (69) in Eq. (68) gives for the Landau damping rate by thermal ions

$$\Gamma_{L}(\theta_{min})\approx \frac{\sqrt{\pi }}{4}\beta^{1/2}{\left(\frac{\epsilon_{d}}{r_{c}v_{A}}\right)}^{1/2},$$ (70)

where *β* ≡ (*v_i_*/*v_A_*)^2^.

Interestingly, Γ_*L*_(*θ_min_*) has the same form as the rate at which cosmic ray generated Alfven waves are sheared apart by fieldline wandering, which is the so-called turbulent damping rate identified in Refs. 30 and 66, but Γ_*L*_(*θ_min_*) is larger by a factor of *β*^1/2^. It should be noted that from Eq. (69), *θ_min_* ∼ (*r_c_*/*L_MHD_*)^1/4^ is much larger than the ratio of *r_c_* to any global scale (such as *L_MHD_* itself). Thus, the damping rate due to obliquity is much larger than found, for example, in Ref. 25, which estimated the damping based on global scales.

Turbulent damping was identified in Ref. 46 as the fastest damping process in galaxy clusters. Preliminary indications are that replacing the turbulent damping rate with the Landau damping rate derived here substantially increases the cosmic ray streaming rate in galaxy clusters, but the quantitative impact on the formation of radio halos, collisionless heating, and *γ*-ray emission have yet to be assessed.

We close on a speculative note that suggests an area for future study. A number of works over the past decade have investigated the possibility of anisotropic thermal pressure in the hotter and more rarefied portions of the ICM (see Ref. 67 for the founding paper and Ref. 68 for a recent study). This work suggests that the ICM is maintained in a state of marginal stability to either the firehose or the mirror instability, depending on which form of anistropy is driven by local expansion or compression of the ambient magnetic field. In a plasma that tends toward firehose instability ($P_{g∥}/P_{g⊥}>1$), the speed of Alfven waves is greatly slowed. It is interesting to speculate upon the effect of this reduced wave speed on cosmic ray transport through the plasma. This is a topic for future work.

## VII. SUMMARY AND OUTLOOK

In this paper we have focussed on when and how cosmic rays, few in number but large in energy density, are collisionlessly coupled to the thermal plasma. This problem is becoming increasingly central to astrophysics with the growing realization that cosmic rays can play an important role in the self-regulation of star formation and supermassive black hole growth in galaxies, i.e., that cosmic rays are a component of feedback.

The cosmic ray coupling problem evolved from studies of particle propagation through fluctuating magnetic fields to discovery of self-confinement through the streaming instability and then to classical cosmic ray hydrodynamics, or CCRH—a fluid theory that macroscopically describes energy and momentum exchange between the cosmic rays and thermal gas, mediated by the gyroresonant streaming instability [Eqs. (4) and (5)]. The main contribution of this paper has been to extend and generalize the fluid theory, so that it can be used in situations where cosmic rays scatter from waves that they do not generate themselves. We call this theory generalized cosmic ray hydrodynamics, GCRH.

The fluid equations for thermal gas, cosmic rays, and waves are summarized in Sec. IV E. In CCRH, cosmic rays are advected down their density gradient at velocity **n***v_A_* relative to the thermal gas. They exert a force $-\nabla P_{c}$ on the gas and deliver heat at the rate $-v_{A}n\cdot \nabla P_{c}$. Diffusion along the magnetic field lines, which depends on the amplitude of the waves that scatter them, is determined by the condition that the waves are marginally stable. In GCRH, the advection velocity relative to the fluid is the intensity weighted mean of co and counter propagating waves [Eqs. (17) and (20)], and vanishes for balanced turbulence. The force on the gas is the same as it is in CCRH, but the heating rate is proportional to the advection rate, so it vanishes for balanced turbulence. Indeed, energy flows from waves to cosmic rays through second order Fermi acceleration, although this is an $O(v_{A}^{2}/c^{2})$ effect.

We showed in Sec. V that the choice of propagation model has a significant impact on problems such as the stability of vertically stratified galactic gas, and the launching of galactic winds. In Sec. VI, we argued the role of cosmic rays in galaxy clusters is also dependent on propagation model.

Many aspects of the subject require additional development, and the outlook is bright. Given all the imponderables in our understanding of interstellar, intracluster, and intergalactic thermodynamics, gas dynamics, and magnetic field structure, it is not easy to say, which if any version of GCRH is the appropriate one in any particular situation. However, that should not deter us from constructing the best possible theories and developing treatments that can be used to model global systems. Generalizing the theory even further to include other wave modes such as magnetosonic waves is important for considerations of energy balance. Including the nonresonant modes is important under extremely high *β* and/or high cosmic ray flux conditions. Experiment has a role to play in studies of particle energization, confinement, and kinetic instabilities.

While the fluid theory presented here probably captures much of the overall energetics, it provides no information about the evolution of the cosmic ray spectrum or about effects such as pressure anisotropy, heat conduction, or viscosity. A full Vlasov theory of cosmic ray interactions is precluded by the vast disparity between the sub solar system cosmic ray gyroscale and the thousands or even millions of light years that characterize galactic and intergalactic scales. However, intermediate frameworks are certainly feasible, such as recent work^69^ that retains spectral information for comparison with observations.

Whatever description of cosmic rays is chosen, we should not neglect mesoscale effects that play out between the kinetic and global scales. Theory and simulation of the Parker and other buoyancy instabilities in light of modern cosmic ray propagation theories may be important for understanding the behavior of gravitationally stratified gas in galaxies. Thermal instability of the cosmic ray heated medium may be equally important for understanding its thermal structure. The modification of shocks and their environments by cosmic rays has been studied for more than three decades, but new effects are constantly discovered.

However fruitful these research directions prove to be, one thing is certain: some of the most important and challenging problems in astrophysics are linked to the plasma physics of cosmic rays.
